# Computed Tomographic Screening Intervals for Patients at Moderate Risk of Lung Cancer

**DOI:** 10.1001/jamanetworkopen.2025.23044

**Published:** 2025-07-24

**Authors:** Koen de Nijs, Harry J. de Koning, Pianpian Cao, Maikol Diasparra, Rochelle Garner, Jihyoun Jeon, Jean H. E. Yong, Rafael Meza, Kevin ten Haaf

**Affiliations:** 1 Department of Public Health, Erasmus University Medical Center Rotterdam, Rotterdam, the Netherlands; 2 Department of Public Health, College of Health and Human Sciences, Purdue University, West Lafayette, Indiana; 3 Statistics Canada, Ottawa, Ontario, Canada; 4 Department of Epidemiology, University of Michigan, Ann Arbor; 5 Canadian Partnership Against Cancer, Toronto, Ontario; 6 BC Cancer Research Institute, Vancouver, British Columbia, Canada; 7 School of Population and Public Health, University of British Columbia, Vancouver, British Columbia, Canada

## Abstract

**Importance:**

The US Preventive Services Task Force (USPSTF) recommends annual computed tomographic (CT) screening for individuals aged 50 to 80 years at high risk of lung cancer. Other countries are issuing similar recommendations, with some opting for biennial screening to reduce the burden of screening. However, it is unknown whether benefits of annual screening can be preserved when adapting the interval to age, sex, and smoking history.

**Objective:**

To evaluate the health outcomes and costs of adaptive lung cancer screening intervals relative to annual screening.

**Design, Setting, and Participants:**

This economic evaluation used comparative modeling methods with 3 models: 2 Cancer Intervention and Surveillance Modeling Network models and the OncoSim model from the Canadian Partnership Against Cancer. Screening of the US 1965 birth cohort with adaptive intervals was evaluated according to age, sex, and smoking exposure. Simulated outcomes are recorded from 2005 to 2065 for subpopulations of 200 000 individuals with smoking history of 10 to less than 20, 20 to less than 30, and 30 or greater pack-years (PY) for each sex. This evaluation was conducted between September 19, 2023, to December 1, 2024.

**Exposure:**

Low-dose regular CT screening among those eligible per USPSTF 2021 recommendations.

**Main Outcomes and Measures:**

Strategy effectiveness was evaluated as lung cancer deaths prevented and life-years gained relative to annual screening. Screening burden is measured by the number of CT screens. To determine cost-effectiveness, quality-adjusted life-years (QALYs) gained and Surveillance, Epidemiology, and End Results– and Medicare-derived costs of treatment were calculated, as well as CT and follow-up examination costs. A willingness-to-pay (WTP) threshold of $100 000/QALY for cost-effectiveness was assumed.

**Results:**

Biennial screening at 50 to 60 years of age, followed by annual screening, reduced CT requirements while preserving most benefits. This strategy preserved 95.9% (intermodel range, 93.5%-97.5%) of lung cancer deaths prevented, compared with annual screening, with 20.6% (intermodel range, 19.3%-21.9%) fewer screens. Annual screening from 50 to 80 years of age was not cost-effective at a WTP threshold of $100 000/QALY. Cost-effective strategies varied by risk group, but all cost-effective strategies started with biennial screening and moved to annual screening at 60 years of age or a PY threshold of 30 to 40 was reached.

**Conclusions and Relevance:**

In this economic evaluation of lung cancer screening, biennial screening for participants younger than 60 years and those with less than 30 PY of smoking exposure maintained screening benefits relative to annual screening. Resource-constricted screening programs may consider adaptive intervals.

## Key Points

**Question:**

Can the benefits of lung cancer screening be preserved while reducing the burden by adapting the annual screening interval to age, sex, and smoking history?

**Findings:**

In this economic evaluation including 3 microsimulation models of lung cancer screening, starting with biennial screening for individuals aged 50 to 60 years preserved most of the benefits (96% of deaths prevented) while requiring 21% fewer computed tomographic screens.

**Meaning:**

These findings suggest that the screening interval may be relaxed to biennial with little loss of benefit for those at a moderate risk of lung cancer, particularly participants aged 50 to 60 years.

## Teaser

This economic evaluation estimates the health outcomes and costs associated with strategies for annual or biennial computed tomographic (CT) screens among patients at moderate risk of lung cancer at different segments of the age range of 50 to 80 years and for subgroups based on sex and smoking exposure.

## Introduction

The National Lung Screening Trial^1^ demonstrated a 20% reduction in lung cancer mortality from annual computed tomographic (CT) screening among high-risk individuals. Following this result, the US Preventive Services Task Force (USPSTF)^2^ recommended screening for those aged 50 to 80 years with greater than 20 pack-years (PY) of exposure. Screening effectiveness was reinforced by results from the NELSON (Dutch-Belgian Lung-Cancer Screening) trial, which showed a 24% reduction in lung cancer mortality.^3^ Regular CT screening allows the detection of lung cancer in an early, resectable stage.^4^ In the absence of screening, lung cancer is often detected in stage IV, when 5-year survival is only 5.8%.^5^

The USPSTF B-recommendation means that many in the US are eligible and insured for screening; modeling estimates suggest that 23.6% of the 1960 cohort are screening eligible.^6^ However, only 17.5% of eligible individuals have undergone screening,^7^ and only 30.5% participated in a repeat screen.^8^ In light of racial disparities incurred by 2013 eligibility criteria, the 2021 USPSTF recommendation expanded eligibility to individuals aged 50 to 54 years and those with 20 to 29 PY of exposure.^2^ A larger screening-eligible population means that more individuals, particularly more African American individuals, may benefit from screening, but also that more resources are needed to achieve full screening coverage.

Meanwhile, other countries are in the phases of assessment or early implementation of lung cancer screening. Outside the US, centralized programs work to document eligibility and encourage screening uptake. These settings may see less difficulty in identifying and reaching individuals eligible for screening to encourage uptake^9,10^ but could face systems-level barriers, such as resource and personnel limitations.^11,12^ For example, the Swiss Cancer Screening Committee issued a recommendation for implementing biennial screening to prevent capacity excesses.^13^ Modeling of screening scenarios showed that CT volume would need to increase 45% nationally to implement annual screening per the 2021 USPSTF guidelines.^14^ Similarly, the province of British Columbia in Canada implemented screening using the International Lung Screening Trial protocol, which recommends a 2-year repeat screen after low-risk screening results, and screened two-thirds of participants.^15^

In light of concerns of repeat screening attendance and capacity management, we evaluated strategies to reduce the screening burden. In particular, strategies with personalized intervals by sex, age, and smoking exposure were considered. The benefits of screening are generally considered proportional to the risk of the disease at the time of screening. A higher risk corresponds to a higher probability of cancer detection, facilitating early intervention. The incurred harms, such as resource requirements, false-positive results, and screening-related anxiety, may, however, be independent of risk. Therefore, less frequent screening may be warranted while at a lower risk, such as for younger individuals or those with a low-to-moderate smoking history.

Previous studies of optimal strategies have applied the screening interval homogenously across risk strata,^16,17^ although 2 recent modeling studies have shown potential benefits of adapting the interval to participant risk.^18,19^ We supplemented this research by using comparative modeling of adaptive lung cancer screening intervals, aggregating the results from 2 models of the US Cancer Intervention and Surveillance Modeling Network (CISNET) Lung Working Group and 1 model developed for Canada. For a 1965 cohort, we estimated the effectiveness of strategies that screen annually or biennially at different segments of the age range 50 to 80 years or for subgroups based on sex and duration of smoking exposure. The strategies were compared by lung cancer outcomes, resource use, and cost incurred per quality-adjusted life-years (QALYs) gained.

## Methods

In this economic evaluation, we used comparative modeling of age-adaptive lung cancer screening strategies with validated microsimulation models from the US and Canada. As a health economic evaluation, no ethical review was needed, and there were no participants requiring informed consent. This study followed the Consolidated Health Economic Evaluation Reporting Standards (CHEERS) guidelines.

Two models were developed as part of the CISNET Lung Working Group and have been used to study lung cancer screening in a variety of settings.^6,14,20,21,22,23^ They were calibrated to the National Lung Screening Trial and Surveillance, Epidemiology, and End Results (SEER) lung cancer outcomes for the US.^24^ Additionally, the Canadian OncoSim-Lung model has been used to evaluate screening effectiveness in Canada.^25^ All models have been included in a comparative modeling study to compare model assumptions and predictions.^26^ Each model conforms to the diagrammatic representation presented in eFigure 1 in Supplement 1. They model individual life histories, for which life-years and disease outcomes are tallied to yield population-level results. Lifetime smoking behavior is modeled, which informs lung cancer onset using smoking dose-response models. The cancer remains undetected during a stochastically generated period. When a screening scenario is simulated, cancer can be detected early at screening events, potentially curing the cancer or prolonging survival. eTable 1 in Supplement 1 gives an overview of model characteristics.

For the US models, we used the CISNET smoking-history generator (SHG) to produce life histories of smoking behavior for the 1965 US birth cohort, including ages of initiation and cessation and cigarettes smoked per day.^27,28^ OncoSim-Lung uses an internal SHG originally developed for Canada, which generates similar smoking exposure as the CISNET SHG (eTable 2 in Supplement 1).

We evaluated lung cancer outcomes with no screening, annual screening, biennial screening, and age-adaptive intervals (Table 1). We stratified results by sex and smoking exposure at 50 years of age (ie, 10 to <20, 20 to <30, and ≥30 PY) to study whether the efficiency of the annual interval varied by risk stratum.

**Table 1.** Lung Cancer Screening Scenarios Studied by Screening Interval Used and Inclusion Criteria^a^

Screening pattern	Age- or PY-specific screening interval
No screening	No screening
Constant interval	
Annual	CT screen every year, aged 50-80 y
Biennial	CT screen every 2 y, aged 50-80 y
Start biennial	
SB10	Biennial, aged 50-59 y; annual, aged 60-80 y
SB15	Biennial, aged 50-64 y; annual, aged 65-80 y
SB20	Biennial, aged 50-69 y; annual, aged 70-80 y
End biennial	Annual, aged 50-69 y; biennial, aged 70-80 y
Intermittent biennial	
MA10	Annual, aged 60-69 y; biennial, aged 50-59 and 70-80 y
MB10	Biennial, aged 60-69 y; annual, aged 50-59 and 70-80 y
Smoking-updated interval	
UB30	Biennial, aged 50-80 y; upgrading to annual when exposure exceeds 30 PY
UB40	Biennial, aged 50-80 y; upgrading to annual when exposure exceeds 40 PY
UB15cess^b^	Annual, aged 50-80 y; changing to biennial when smoking cessation exceeds 15 y

Abbreviations: CT, computed tomographic; PY, pack-years.

^a^ All strategies assumed screening eligibility as per 2021 US Preventive Services Task Force (USPSTF) recommendations to have smoked at a minimum of 20 PY and not to have quit smoking longer than 15 years ago. The lowest risk stratum (10 to <20 PY at 50 years of age) was assumed to only start screening when they first exceed 20 PY.

^b^ As a sensitivity analysis, we also ran each strategy without the 2021 USPSTF inclusion criterion of a maximum of 15 years since smoking cessation, per recommendations from the American Cancer Society.^29^ Only this sensitivity analysis included strategy UB15cess.

The primary outcomes studied were the number of CT screens required relative to the life-years gained and deaths prevented due to screening. Additionally, we noted for each scenario the lung cancer incidence, lung cancer deaths, and the life-years lived as well as screening-induced harms such as overdiagnosis, false-positive results, and unnecessary biopsies. Cost values of screening, diagnosis, and treatment were also included to facilitate economic evaluation of the strategies from a US health care sector perspective. Cost and health utility inputs are reported in eTables 3 and 4 in Supplement 1 and match values previously used in CISNET modeling studies.^20,30^ This includes health disutilities associated with undergoing screening and receiving indeterminate results. Treatment costs were derived from SEER-Medicare data,^31^ and health-related quality of life was adapted from EQ-5D surveys among patients with lung cancer.^32^ Cost values were adjusted to the 2023 price index.^33^ The time horizon extends to 2065, and total costs and QALYs were discounted at 3%.^34^ We reported the strategies on the efficient frontier (those that gain the most QALYs for a given cost level) and gave the incremental cost-effectiveness ratio (ICER), that is, the extra cost incurred for the strategy, relative to the previous strategy on the frontier.^34^

As the base case, we assumed USPSTF eligibility requirements, including the exclusion of individuals with greater than 15 years since smoking cessation. However, recent evidence suggests that screening may be warranted beyond this threshold.^22,29^ We therefore additionally simulated outcomes when we changed the baseline from USPSTF eligibility to the American Cancer Society (ACS) guidelines,^29^ including all those aged 50 to 80 years with greater than 20 PY of exposure regardless of cessation years. In this analysis, we added a strategy that screened annually until greater than 15 years since smoking cessation and continued with biennial screening thereafter (UB15cess).

We assumed screening uptake and adherence to be 100% in the simulated scenarios. Although this idealized the state of lung cancer screening, it was the optimal method for determining the most efficient screening strategy. Lower assumed uptake would overprescribe screening to counter uptake effects.^35^ Our results should, therefore, be interpreted as the expected benefits and harms for the individual attending screening rather than the projected scale of population-level effects.

### Statistical Analysis

This analysis was conducted between September 19, 2023, and December 1, 2024. Model results were collected from each institute using homogenized results templates. Unless stated otherwise, outcomes are reported as the mean across models. The intermodel range (IMR) is shown in graphs to quantify intermodel variation. Data aggregation and analysis were performed using Excel, version 2408 (Microsoft Corporation).

## Results

We present our results in order of the complexity of strategies studied. First, we compared annual screening with age-adaptive screening by the CT burden, deaths prevented, and life-years gained. Second, we showed screening effectiveness when age-adaptive schedules were applied by sex and smoking exposure stratum. Finally, we performed an economic evaluation, considering cost per QALY of age-adaptive screening relative to annual and biennial screening.

### Age-Adaptive Screening

The benefits and harms of annual screening, biennial screening, and adaptive screening strategies (applied equally to all screening-eligible individuals) are presented in Table 2. To show intermodel variability, the results from each of the 3 natural history microsimulation models are reported in eTable 7 in Supplement 1.

**Table 2.** Summary of Benefits and Harms of Age-Adaptive Screening Strategies Across 3 Microsimulation Models^a^

Strategy^b^	CT scans		Lung cancer					Life-Years		No. of excess procedures		No. of lung cancer cases	Overdiagnosis rate, %^e^	No. of CT screens/death prevented
	No. of scans	Reduction, %^c^	No. of screen-detected cases	No. of clinical cases	No. of deaths	No. of deaths prevented^d^	Fewer deaths, %^c^	No. gained^d^	Reduction, %^e^	Follow-up CT scans	Biopsies			
No screening	0	100	NA	18 250	11 611	NA	100	NA	100	NA	NA	18 250	0	NA
Annual (reference)	1 264 000	0	6215	12 405	9224	2387	0	40 496	0	66 344	4417	18 620	5.9	530
Biennial	651 000	48.5	5011	13 505	9773	1838	23.0	31 009	23.4	37 060	2672	18 516	5.3	354
SB10	1 004 000	20.6	6015	12 593	9323	2288	4.1	37 827	6.6	53 746	3755	18 608	6.0	439
SB15	888 000	29.8	5835	12 755	9402	2209	7.5	36 000	11.1	48 821	3436	18 590	5.8	402
SB20	778 000	38.5	5577	12 994	9533	2078	12.9	33 855	16.4	42 999	3096	18 571	5.8	374
EB10	1 137 000	10.1	5672	12 901	9462	2150	9.9	37 687	6.9	60 445	3993	18 573	5.7	529
MA10	877 000	30.7	5463	13 088	9564	2048	14.2	34 922	13.8	47 781	3312	18 551	5.5	428
MB10	1 038 000	17.9	5788	12 793	9422	2189	8.3	36 660	9.5	55 671	3772	18 581	5.7	474
UB30	1 051 000	16.9	5977	12 627	9339	2272	4.8	38 494	4.9	56 037	3849	18 604	5.9	462
UB40	888 000	29.8	5766	12 816	9430	2181	8.6	36 636	9.5	48 210	3396	18 583	5.8	407

Abbreviations: CT, computed tomography; NA, not applicable.

^a^ Strategies are applied to all individuals meeting 2021 US Preventive Services Task Force criteria (exposure of 20 pack-years [PY] and maximum of 15 years since smoking cessation). The mean of the 3 microsimulation models is shown, with model-specific results reported in eTable 7 in Supplement 1. Outcomes are scaled per 100 000 individuals alive at 45 years of age, with a minimum smoking exposure of 10 PY at 50 years of age (some of whom may become eligible at a later age as they accumulate PY).

^b^ Given in Table 1.

^c^ Compared with annual screening.

^d^ Compared with no screening.

^e^ Indicates overdiagnosed lung cancer cases, given by the proportion of screen-detected lung cancer that would not have presented clinically in a scenario without screening.

Tailoring screening intervals to become more frequent with age (when risk increases) maintained most of the benefits of annual screening but required fewer scans. Starting with 10 to 20 years of biennial screening at 50 to 59 years of age (strategies SB10, SB15, and SB20 in Table 2) reduced CT scans by 20.6% (IMR, 19.3%-21.9%) to 38.5% (IMR, 37.4%-39.5%) but maintained 87.1% (IMR, 82.6%-90.9%) to 95.9% (IMR, 93.5%-97.5%) of lung cancer deaths prevented and 83.6% (IMR, 78.7%-88.0%) to 93.4% (IMR, 90.6%-95.6%) of life-years gained, as compared with annual screening. For instance, for SB15, this reduced the number of screens per death prevented from 530 (IMR, 417-679) for annual screening to 402 (IMR, 308-527). Other patterns of age-adaptive screening, such as ending with biennial screening, were less efficient.

We also tailored the interval to smoking exposure measured by PY, rather than age only. These strategies started with biennial screening and switched to annual screening when the individual reached 30 or 40 PYs of exposure (strategies UB30 and UB40, respectively). We found UB30 and UB40 to be more efficient than annual screening, with 462 (IMR, 360-603) and 407 (IMR, 318-530) screens per death prevented, respectively, compared with 530 for annual screening. UB30 and UB40 would reduce CT volume relative to annual screening by 16.9% (IMR, 16.6%-17.4%) and 29.8% (IMR, 29.1%-30.2%), respectively.

### Efficient Strategies by Risk Stratum

To further personalize the interval, age-adaptive strategies were implemented based on individual PY of exposure, offering someone with lower smoking exposure a sparse screening schedule. The Figure shows reductions relative to annual screening of CT burden and deaths prevented for risk groups of 10 to less than 20, 20 to less than 30, and 30 or greater PY (at 50 years of age). Starting with biennial screening preserved screening benefits for each group and reduced screening burden. This reduction was highest among those with exposure of 20 to less than 30 PY. Strategies that adapted the interval to the smoking exposure (UB30 and UB40) reduced the CT screening burden among those with moderate exposure, but also reduced the benefits of screening to close to those of biennial screening. eFigure 2 in Supplement 1 demonstrates the same result for life-years gained with adaptive strategies, which were reduced at a much lower rate relative to the reduction in the screening burden.

**Figure.**
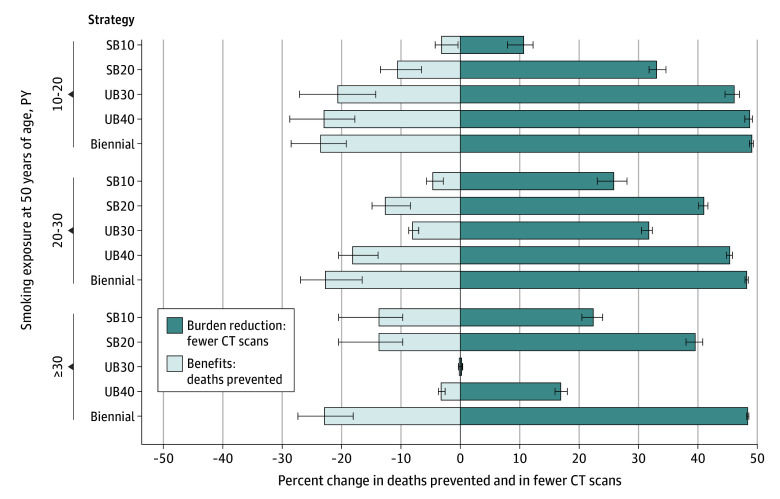
Change in Deaths Prevented and Reduction in Computed Tomography (CT) Burden Relative to Annual Screening for Biennial and Age-Adaptive Screening Screening strategies consist of 10 to 20 years of biennial screening at 50 to 69 years of age (SB10 and SB20) or age-adaptive screening starting with biennial screening and switch to annual screening when the individual reaches 20 to 40 PYs of exposure (UB20, UB30, and UB40). Error bars represent the intermodel range of the results across the 3 microsimulation models included in our study. This Figure pertains to men and women with increasing lung cancer risk categories at 10 to less than 20, 20 to less than 30, and 30 or more pack-years (PY) of exposure at 50 years of age. Those with exposure of 10 to less than 20 PY start screening if they cross the 2021 USPSTF threshold of 20 PY. The full input data to this figure, as well as the data for strategies not shown here, are presented in eTable 5 in Supplement 1.

eTable 5 in Supplement 1 shows these outcomes stratified also by sex. For most strategies, women saw a lower drop in screening benefits when moving from annual screening to adaptive intervals. For example, UB30 reduced CT volume among those with exposure of 20 to less than 30 PY by 30.5% (IMR, 30.0%-31.5%) for women and 32.9% (IMR, 31.0%-34.4%) for men. Deaths prevented and life-years gained were reduced by 7.3% (IMR, 6.8%-8.3%) and 9.4% (IMR 8.6%-11.8%) for women, respectively, and 9.2% (IMR, 8.1%-10.7%) and 10.8% (IMR, 8.8%-13.1%) for men, respectively.

### Cost-Effectiveness of Age-Adaptive Screening by Risk Stratum

To weight the cost savings of adaptive intervals against the reduction in benefits, we evaluated cost per QALY gained for each strategy. Table 3 shows the preferred strategies by risk group, assuming increasing levels of WTP threshold for a QALY. Overall, adaptive screening strategies were the most efficient strategies to a WTP threshold of $100 000. The efficient strategies varied by risk group, but for most groups it was preferred either to start with biennial screening for 10 or 15 years or to screen biennially until 30 or 40 PY of exposure were reached. Annual screening was cost-effective when we increased the WTP to $150 000 or $200 000.

**Table 3.** Optimal Age-Adaptive Screening Strategies From the Efficient Frontier at Increasing WTP Thresholds by Risk Stratum and Biological Sex^a^

Risk stratum, PY at 50 y of age	Optimal screening strategy by WTP (cost/QALY threshold)				
	$50 000	$75 000	$100 000	$150 000	$200 000
Men					
10 to <20	UB40 ($46 583)	UB30 ($64 265)	UB30 ($64 265)	Annual ($146 267)	Annual ($146 267)
20 to <30	No screening^b^	UB40 ($57 821)	SB15 ($96 094)	SB10 ($140 505)	SB10 ($140 505)
≥30	Biennial ($35 826)	SB10 ($70 640)	UB40 ($95 474)	UB40 ($95 474)	Annual ($156 008)
All	Biennial ($40 809)	UB40 ($66 779)	UB40 ($66 779)	UB30 ($145 187)	Annual ($188 997)
Women					
10 to <20	Biennial ($40 105)	UB30 ($68 295)	SB20 ($96 371)	SB10 ($122 392)	SB10 ($122 392)
20 to <30	UB40 ($47 284)	SB20 ($61 291)	SB10 ($80 329)	SB10 ($80 329)	SB10 ($80 329)
≥30	SB15 ($49 994)	UB40 ($71 042)	UB40 ($71 042)	Annual ($121 349)	Annual ($121 349)
All	Biennial ($31 934)	UB40 ($51 325)	UB40 ($51 325)	Annual ($148 016)	Annual ($148 016)
Both					
10 to <20	Biennial ($45 692)	UB30 ($64 841)	UB30 ($64 841)	SB10 ($131 668)	Annual ($170 744)
20 to <30	Biennial ($45 478)	SB20 ($74 638)	SB10 ($95 173)	SB10 ($95 173)	SB10 ($95 173)
≥30	SB20 ($48 568)	SB10 ($63 257)	UB40 ($84 870)	Annual ($135 412)	Annual ($135 412)
All	Biennial ($35 886)	UB40 ($58 915)	UB40 ($58 915)	UB30 ($123 379)	Annual ($162 857)

Abbreviations: PY, pack-years; QALY, quality-adjusted life-year; WTP, willingness to pay.

^a^ The cost-efficient age-adaptive lung cancer screening strategy at increasing WTP thresholds and the incremental cost-effectiveness ratio (ICER; the incremental cost per QALY gained) of that strategy relative to the strategy preceding it on the efficient frontier (the most efficient strategy yielding fewer total QALYs) are shown for different strata. Strategies are described in Table 1. For the lowest risk stratum, some may start screening as they cross the 20-PY minimum exposure set by the 2021 US Preventive Strategy Task Force guidelines, while some may never be screened. Across all risk strata, individuals may leave screening as they cross 15 years since smoking cessation.

^b^ Indicates that no strategy studied had an ICER below the WTP threshold.

For the entire screening population, eTable 6 in Supplement 1 shows the cost-effectiveness of all strategies studied. We found that relative to no screening, all strategies were cost-effective in their costs per QALY. However, compared with age-adaptive screening (UB30), annual screening for individuals aged 50 to 80 years had an ICER of $162 857/QALY, as opposed to $46 576/QALY relative to no screening. That is, annual screening per USPSTF criteria was a cost-effective intervention by itself, but more efficient alternatives were given by the adaptive schedules.

Strategies UB30 and UB40 would require continuous assessment of duration of exposure to determine the interval. If this is not feasible in practice, age-adaptive schedules that are set at entry to screening may be preferred. eTable 8 in Supplement 1 shows the frontiers if we excluded UB30 and UB40 from consideration, focusing only on the age-adaptive schedules. In this case, SB10 was preferred for most groups at a WTP threshold of $100 000. Annual screening was only preferred population-wide from a WTP of approximately $146 000/QALY.

As Table 3 shows, the efficient strategies for increasing WTP depended on the risk group. Annual screening incurred the lowest cost per QALY for those with exposure of 30 PY or greater, of $121 349 for women and $156 008 for men. For those with exposure of 20 to 30 PY, annual screening was not cost-effective even at $200 000/QALY. For those with exposure of 10 to 20 PY, annual screening did appear on the efficient frontier, at a cost of $170 744 for the entire population. Because of their lower smoking exposure, this group may contain many who will become ineligible within the 50- to 80-year age bracket per the smoking cessation limit of 15 years set by the USPSTF. As a consequence, annual screening did not account for 30 years of screening for this group.

eFigure 3 in Supplement 1 shows the cost-QALY plane for each stratum, showing that the age-adaptive strategies varied most in efficiency for those with exposure of 20 to 30 PY. The cost-effectiveness of the annual interval was also the lowest for this group, given by the shallow slope of the efficient frontier from SB10 to annual screening, which corresponded with few QALYs gained for a large increase in cost.

### Outcomes When Relaxing the Threshold of 15 Years Since Smoking Cessation 

eTable 9 in Supplement 1 reports summary statistics of the strategies studied when we relaxed the USPSTF threshold of years since smoking cessation, equivalent to ACS guidelines. Similar to the scenario with the 2021 USPSTF guideline as a baseline, we found improved efficiency from starting with biennial screening: SB10 reduces CT volume by 17%, for 3.4% fewer deaths prevented and 5.8% fewer life-years gained, reducing the CT screens per death prevented from 586 (with annual screening) to 504. The economic evaluation under the ACS guidelines is summarized in eTable 10 in Supplement 1. We found annual screening was only cost-effective for women with exposure of 30 or more PY, from $131 460/QALY WTP; for other groups, strategies UB30 and UB40 were preferred.

## Discussion

In this economic evaluation, we assessed the outcomes associated with adjusting screening intervals based on individuals’ lung cancer risk, exploring less frequent screening options than recommended by the current USPSTF guidelines. Our results identified subgroups of individuals at moderate risk of lung cancer, for whom less frequent screening may preserve most of the benefits. For resource-constrained screening programs, this may be a suitable alternative to annual screening as well as for individual screening of participants who may prefer less frequent screening. Annual screening from 50 to 80 years of age was found to be cost-effective relative to no screening, but not when compared with age-adaptive intervals. When screening biennially for 10 to 15 years or until exposure of 30 or 40 PY was reached, 16.9% to 38.5% of CT screens were reduced. Policymakers could consider these as alternatives to annual screening, considering also the system-specific feasibility of adaptive scheduling.

Previous effectiveness studies have predominantly considered the screening interval to be independent of age or lung cancer risk.^16^ A recent study compared age-adaptive screening to the theoretical optimal screening schedule generated by the ENGAGE (Individualized Lung Cancer Screening Decisions) framework.^18^ It found that starting with biennial screening would be the closest approximation of the optimal schedule. Similar results were found in an analysis using a threshold method to determine optimal screening schedules based on an individual’s previous screening results, lung cancer risk, and life expectancy, which also evaluated the performance of adaptive strategies across PY groups.^19^ We expanded on these single-model results by controlling for model variability through a 3-model comparative analysis. Our analysis also builds on previous studies by showing that age-specific PY-based exposure is also a determinant of the efficiency of the annual interval. We also considered age-adaptive screening relative to ACS guidelines, including alternative frequencies once individuals exceed 15 years since smoking cessation.

In 2021, the USPSTF expanded screening to those with exposure of 20 to 29 PY and lowered the starting age from 55 to 50 years. This was found to reduce screening inequities^36^ but may also reduce efficiency by exposing more individuals at lower risk to annual screening. To balance benefits and harms, such as the cost and capacity management of screening, as well as the burden of false-positive results and overdiagnosis, it may be recommendable to reduce the frequency of screening for individuals at moderate risk while preserving the equity benefit of widespread screening. Future cohorts will increasingly be composed of people with low to moderate smoking exposure.^37^ These individuals have a lower risk for lung cancer than currently eligible cohorts but also a higher life expectancy. Sparser screening schedules may be a suitable alternative for this moderate-risk group.

### Limitations

We note a few limitations to our study, which may be addressed by future research. We modeled the effects for a single 1965 birth cohort, while the lung cancer risk profile will be different for more recent cohorts. In further stratification of the screening interval, we also have not yet accounted for past screening results, which also communicate lung cancer risk.^38,39^ Initially indeterminate screening results that require further follow-up were associated with a higher risk of lung cancer than negative screening outcomes. Effectiveness estimates of further adaptation of the interval should incorporate results from the ongoing 4-IN-THE-LUNG-RUN (Toward Individually Tailored Invitations, Screening Intervals, and Integrated Comorbidity Reducing Strategies in Lung Cancer Screening) trial,^40^ which studies the noninferiority of biennial screening for participants with negative screening outcomes at baseline. Risk-based selection into screening, for example with the PLCOm2012 (Prostate, Lung, Colorectal, and Ovarian 2012 modified model) multivariate risk score,^41^ has been shown to be more efficient at selecting screening participants than PY-based criteria.^14,20^ It is not known whether our results extend to a risk-based screening cohort, where typically screening starts beyond 50 years of age, only when the risk threshold is reached. Future research may consider whether this extension mitigates the benefits of adaptive intervals, by instead adapting the starting age of screening. Last, we were limited in scope in this study by not including sensitivity analyses of individual cost and QALY inputs, which have previously been shown for lung cancer screening by members of our group.^20,30^

## Conclusions

In this economic evaluation study of lung cancer screening, we found that the annual screening interval may be associated with a burden of screening that warrants biennial screening for those at lower risk. Starting with 10 to 15 years of biennial screening would preserve much of the benefits of annual screening while reducing screening-related costs and harms. This result applies particularly to women and those with exposure of 20 to 29 PY of smoking at 50 years of age. Resource-constricted screening programs might want to consider adaptive intervals.

## Notes

Supplement 1. **eTable 1.** Model Descriptions **eTable 2.** Comparison of CISNET and OncoSim Smoking History Generator Outputs **eTable 3.** Health-Related Quality of Life Model Inputs **eTable 4.** Cost Inputs **eTable 5.** Reduction in CT Scan Burden and Benefits of Screening When Reducing From Annual to Age-Adaptive Strategies **eTable 6.** Financial Cost, QALY, and LY Gained of All Strategies **eTable 7.** Benefits and Harms of Age-Adaptive Screening Strategies **eTable 8.** Cost-Effective Age-Adaptive Screening Strategies at Increasing WTP Thresholds by Risk Stratum (in Pack-Years at 50 Years of Age) and Biological Sex, Excluding Strategies That Require Updated PY Information **eTable 9.** Benefits and Harms of Age-Adaptive Screening Strategies, Assuming American Cancer Society Eligibility **eTable 10.** Cost-Effective Age-Adaptive Screening Strategies at Increasing WTP Thresholds by Risk Stratum and Biological Sex, per American Cancer Society Guidelines **eFigure 1.** Model Structure Diagram **eFigure 2.** Life-Years Gained and Reduction in CT Scans Required for Annual, Biennial, and Age-Adaptive (Starting With Biennial) Screening **eFigure 3.** Cost-Effective Frontier of Age-Adaptive Screening Strategies by Sex and Risk Stratum
eReferences.

Supplement 2.
Data Sharing Statement
