# Supplementary material for: Computed Tomographic Screening Intervals for Patients at Moderate Risk of Lung Cancer
Source: JAMA Netw Open. 2025 Jul 24;8(7):e2523044. doi: 10.1001/jamanetworkopen.2025.23044 (PMC12290729; doi:10.1001/jamanetworkopen.2025.23044)
Supplement: Supplement 1. — eTable 1. Model Descriptions eTable 2. Comparison of CISNET and OncoSim Smoking History Generator Outputs eTable 3. Health-Related Quality of Life Model Inputs eTable 4. Cost Inputs eTable 5. Reduction in CT Scan Burden and Benefits of Screening When Reducing From Annual to Age-Adaptive Strategies eTable 6. Financial Cost, QALY, and LY Gained of All Strategies eTable 7. Benefits and Harms of Age-Adaptive Screening Strategies eTable 8. Cost-Effective Age-Adaptive Screening Strategies at Increasing WTP Thresholds by Risk Stratum (in Pack-Years at 50 Years of Age) and Biological Sex, Excluding Strategies That Require Updated PY Information eTable 9. Benefits and Harms of Age-Adaptive Screening Strategies, Assuming American Cancer Society Eligibility eTable 10. Cost-Effective Age-Adaptive Screening Strategies at Increasing WTP Thresholds by Risk Stratum and Biological Sex, per American Cancer Society Guidelines eFigure 1. Model Structure Diagram eFigure 2. Life-Years Gained and Reduction in CT Scans Required for Annual, Biennial, and Age-Adaptive (Starting With Biennial) Screening eFigure 3. Cost-Effective Frontier of Age-Adaptive Screening Strategies by Sex and Risk Stratum eReferences. [file jamanetwopen-e2523044-s001.pdf]

Supplementary Online Content

de Nijs K, de Koning HJ, Cao P, et al. Computed tomographic screening intervals for patients at moderate risk of lung cancer. *JAMA Netw Open.* 2025;8(7):e2523044. doi:10.1001/jamanetworkopen.2025.23044

eTable 1. Model Descriptions

eTable 2. Comparison of CISNET and OncoSim Smoking History Generator Outputs

eTable 3. Health-Related Quality of Life Model Inputs

eTable 4. Cost Inputs

eTable 5. Reduction in CT Scan Burden and Benefits of Screening When Reducing From Annual to Age-Adaptive Strategies

eTable 6. Financial Cost, QALY, and LY Gained of All Strategies

eTable 7. Benefits and Harms of Age-Adaptive Screening Strategies

eTable 8. Cost-Effective Age-Adaptive Screening Strategies at Increasing WTP Thresholds by Risk Stratum (in Pack-Years at 50 Years of Age) and Biological Sex, Excluding Strategies That Require Updated PY Information

eTable 9. Benefits and Harms of Age-Adaptive Screening Strategies, Assuming American Cancer Society Eligibility

eTable 10. Cost-Effective Age-Adaptive Screening Strategies at Increasing WTP Thresholds by Risk Stratum and Biological Sex, per American Cancer Society Guidelines

eFigure 1. Model Structure Diagram

eFigure 2. Life-Years Gained and Reduction in CT Scans Required for Annual, Biennial, and Age-Adaptive (Starting With Biennial) Screening

eFigure 3. Cost-Effective Frontier of Age-Adaptive Screening Strategies by Sex and Risk Stratum

eReferences.

This supplementary material has been provided by the authors to give readers additional information about their work.

© 2025 de Nijs K et al. *JAMA Network Open*.

eTable 1. Model Descriptions

Model Components	Erasmus MISCAN	BCCRI-LungCan	Oncosim
Smoking History Generator	CISNET	CISNET	Internal
Type of smoking dose-response model	two-stage clonal expansion model	two-stage clonal expansion model	The impact of smoking on lung cancer incidence is non-linear and acts through smoker type, intensity, and duration.
Datasets used for dose-response parameter calibration	NHS/HPFS, SEER, NLST, PLCO by sex	NHS/HPFS, U.S. mortality by sex	N/A
Histological types	Adenocarcinoma+BAC+ large cell, squamous, SCLC, ONSCLC<br>Distribution of histologies differs by sex	Adenocarcinoma+BAC, squamous, SCLC, other as a function of sex, smoking status, cigarettes per day, smoking duration, and years since quitting	Non-small cell lung cancer (NSCLC) and small cell lung cancer (SCLC)
Lung cancer stages	IA, IB, II, IIIA, IIIB, IV	IA, IB, II, IIIA, IIIB, IV	NSCLC cases are staged as I, II, IIIA, IIIB, and IV<br><br>SCLC cases are classified as limited (stages I, II, or III) or extensive (stage IV)
Stage progression	Markov state-transition by histology and sex	Markov state-transition by histology and sex	Apart from recurrence, stage progression is not modelled.
Lung cancer survival	By sex, histology, and stage; based on SEER 18 2004-2010	By sex, histology, stage, and age at diagnosis; based on SEER 18 2005-2012	Weibull survival curves depend on cancer stage and treatment path. Further impacts on survival are assessed through relative risks due to: screening, Canadian region, age, sex, treatment, and period effects.
Other-cause mortality	U.S. rates (NCI Smoking History Generator) by sex	Gompertz model of OC mortality calibrated to each trial by sex<br><br>U.S. rates (NCI Smoking History Generator) by sex	Canadian rates according to Vital Statistics by sex
Screening sensitivity model	By stage and histology	By stage, histology, and sex	Screening round, age group and NLST trial data; only NSCLC is detectable by screening in the model
Screening effectiveness	Cure model[a]	Stage-shift model[b]	Screening results in stage shift and lower mortality risk (implemented through relative risk)
Positive nodule follow-up algorithm	Implicit based on the NLST	Implicit based on Lung-RADS	The user can choose between Lung-RADS and NLST. This analysis used NLST

[a] The benefit of screening is based on a stage-specific cure rate.
[b] The benefit of screening is based on an earlier stage at detection relative to clinical detection.

Abbreviations: BAC=bronchioloalveolar carcinoma; CISNET=Cancer Intervention and Surveillance Modeling Network; Lung-RADS=Lung Imaging Reporting and Data System; MISCAN=Microsimulation Screening Analysis; BCCRI-LungCan=BC Cancer Research Institute Lung Cancer (previously known as the University of Michigan Model); NCI=National Cancer Institute;

© 2025 de Nijs K et al. *JAMA Network Open*.

NHS/HPFS=Nurses' Health Study/Health Professionals' Followup Study; NLST=National Lung Screening Trial; OC=other-cause; ONSCLC=other non-small cell lung cancers; PLCO=Prostate, Lung, Colorectal, and Ovarian Cancer Screening Trial; SCLC=small-cell lung cancer; SEER=Surveillance, Epidemiology, and End Results.

© 2025 de Nijs K et al. *JAMA Network Open*.

eTable 2. Comparison of CISNET and OncoSim Smoking History Generator Outputs

Sex	Smoking exposure stratum in packyears (PY) at age 50	Average PY At age 50		At age 60		At age 70		Average PLCOm 2012 (6-year risk) At age 50		At age 60		At age 70	
		OncoSim	CISNET	OncoSim	CISNET	OncoSim	CISNET	OncoSim	CISNET	OncoSim	CISNET	OncoSim	CISNET
Female	[10, 20) PY	14.30	14.63	16.83	16.76	18.31	17.91	0.21%	0.25%	0.49%	0.55%	1.05%	1.07%
Female	[20, 30) PY	24.69	24.35	28.38	26.63	30.48	27.85	0.47%	0.47%	1.08%	0.94%	2.24%	1.74%
Female	[30, ∞) PY	35.61	38.65	42.62	45.83	46.72	49.70	0.73%	0.84%	1.91%	1.94%	4.28%	3.89%
Male	[10, 20) PY	14.46	14.57	16.43	16.50	17.32	17.49	0.22%	0.25%	0.46%	0.52%	0.86%	1.00%
Male	[20, 30) PY	24.62	24.39	27.80	26.45	29.38	27.47	0.47%	0.45%	0.98%	0.88%	1.85%	1.59%
Male	[30, ∞) PY	38.32	41.20	46.20	48.47	50.97	52.21	0.78%	0.85%	1.99%	1.93%	4.33%	3.81%

Supplementary Table 2 summarizes the smoking exposure of smoking histories drawn from both the OncoSim internal smoking history generator, and the common CISNET smoking history generator. For the six risk strata used in this analysis, all from a 1965 birth cohort, mean PY and PLCOm2012 risk at ages 50, 60 and 70 are reported.

© 2025 de Nijs K et al. *JAMA Network Open*.

eTable 3. Health-Related Quality of Life Model Inputs

Input	Category	Value	Source
Population reference health utility	Males 40-49	0.881	Hanmer et al. (2006)[1]
	Males 50-59	0.854	
	Males 60-69	0.841	
	Males 70-79	0.822	
	Males 80+	0.752	
	Females 40-49	0.868	
	Females 50-59	0.838	
	Females 60-69	0.824	
	Females 70-79	0.786	
	Females 80+	0.747	
Disease-specific health utility	Stage I NSCLC	0.71	Tramontano et al. (2015)[2] and McMahon et al. (2011)[3]
	Stage II NSCLC	0.68	
	Stage III NSCLC	0.67	
	Stage IV NSCLC	0.66	
	Limited SCLC	0.69	
	Extensive SCLC	0.66	
	Terminal LC	0.62	
Screening-related loss of quality of life	Screening LDCT	-8.75 hours	Caverly et al. (2018)[4]
	Indeterminate Finding	-0.01	Toumazis et al. (2019) [4, 5]

© 2025 de Nijs K et al. *JAMA Network Open*.

eTable 4. Cost Inputs

	Treatment Cost in 1000's of USD ($) per person-year from age[6]:									
Category[#]	45	50	55	60	65	70	75	80	85	90
SCLC Lim. Initial*	120.1	120.1	120.1	106.0	91.9	81.1	70.3	62.1	53.8	53.8
SCLC Lim. Cont.†	39.2	39.2	39.2	32.3	25.4	21.0	16.5	13.6	10.7	10.7
SCLC Lim. Term-LC‡	162.6	162.6	162.6	145.6	128.6	115.2	101.7	91.1	80.5	80.5
SCLC Lim. Term-OC§	111.8	111.8	111.8	101.7	91.6	83.3	75.0	68.2	61.4	61.4
SCLC Ext. Initial*	120.0	120.0	120.0	108.1	96.2	86.6	77.1	69.4	61.8	61.8
SCLC Ext. Cont.†	46.1	46.1	46.1	41.5	36.9	33.3	29.6	26.7	23.7	23.7
SCLC Ext. Term-LC‡	177.4	177.4	177.4	169.5	161.6	154.4	147.2	140.7	134.1	134.1
SCLC Ext. Term-OC§	160.5	160.5	160.5	149.5	138.5	129.0	119.5	111.4	103.2	103.2
NSCLC(St. I) Initial*	84.4	84.4	84.4	77.4	70.4	64.5	58.7	53.8	48.9	48.9
NSCLC(St. I) Cont.†	12.0	12.0	12.0	11.7	11.5	11.2	11.0	10.7	10.5	10.5
NSCLC(St. I) Term-LC‡	144.4	144.4	144.4	129.9	115.4	103.8	92.2	82.9	73.7	73.7
NSCLC(St. I) Term-OC§	131.2	131.2	131.2	117.7	104.1	93.4	82.6	74.1	65.6	65.6
NSCLC(St. II) Initial*	138.2	138.2	138.2	124.4	110.5	99.4	88.3	79.5	70.6	70.6
NSCLC(St. II) Cont.†	13.3	13.3	13.3	13.3	13.3	13.3	13.3	13.3	13.3	13.3
NSCLC(St. II) Term-LC‡	179.1	179.1	179.1	160.5	141.9	127.2	112.5	100.8	89.1	89.1
NSCLC(St. II) Term-OC§	119.9	119.9	119.9	107.1	94.4	84.3	74.3	66.4	58.5	58.5
NSCLC(St. III) Initial*	134.4	134.4	134.4	117.5	100.6	87.9	75.3	65.8	56.3	56.3
NSCLC(St. III) Cont.†	46.2	46.2	46.2	39.1	32.0	27.1	22.2	18.8	15.4	15.4
NSCLC(St. III) Term-LC‡	194.7	194.7	194.7	173.6	152.4	135.9	119.4	106.4	93.5	93.5
NSCLC(St. III) Term-OC§	123.9	123.9	123.9	112.9	102.0	93.0	83.9	76.5	69.1	69.1
NSCLC(St. IV) Initial*	140.3	140.3	140.3	121.6	103.0	89.3	75.6	65.6	55.5	55.5
NSCLC(St. IV) Cont.†	57.8	57.8	57.8	51.3	44.7	39.6	34.6	30.6	26.7	26.7
NSCLC(St. IV) Term-LC‡	234.7	234.7	234.7	211.7	188.7	170.2	151.8	136.9	122.0	122.0
NSCLC(St. IV) Term-OC§	223.9	223.9	223.9	196.3	168.8	148.0	127.2	111.6	95.9	95.9

Category	Item cost in USD ($) per event[7]:
Chest Radiograph	26.27
LDCT Screening	319.46
LDCT follow-up	285.14
Bronchoscopy	425.81
Mediastinoscopy	381.32
Needle Biopsy	471.57
VATS	462.67
PET/CT	1659.99

*: Initial care, the first 6 months of treatment, †: Continuing care, the period of treatment between initial and terminal care, up to 5 years, ‡: Terminal care for those with lung cancer dying from lung cancer (LC), §: Terminal care for those with lung cancer dying from other causes (OC). #: Lung cancer care costs per person-year are stratified by the histological type (Small-cell Lung Cancer [SCLC] and non-small cell lung cancer[NSCLC]) and stage [Limited and Extended for SCLC and TNM stage I, II, III and IV for NSCLC]). Values adjusted to 2023 U.S. Consumer price index levels.[8]

© 2025 de Nijs K et al. *JAMA Network Open*.

eTable 5.– Reduction in CY scan burden and benefits of screening when reducing from annual to age-adaptive strategies,

		Female			Male		
Risk Stratum	Strategy	CT reduction	Deaths prevented reduction	LY Gained reduction	CT reduction	Deaths prevented reduction	LY Gained reduction
10-20 PY	Annual	0.0% [0.0-0.0]	0.0% [0.0-0.0]	0.0% [0.0-0.0]	0.0% [0.0-0.0]	0.0% [0.0-0.0]	0.0% [0.0-0.0]
	EB10	17.0% [15.2-17.9]	12.7% [10.3-15.4]	10.4% [8.9-12.6]	15.3% [14.1-17.1]	11.6% [8.7-15.5]	8.8% [5.8-11.4]
	MA10	27.1% [25.7-28.2]	13.8% [11.5-16.6]	12.2% [11.2-14.8]	26.7% [25.3-28.0]	15.2% [11.1-18.0]	14.0% [10.8-16.0]
	MB10	22.2% [21.1-23.6]	7.4% [6.0-10.0]	10.1% [8.8-13.0]	22.7% [21.5-24.1]	8.5% [7.2-9.8]	10.9% [10.0-13.2]
	SB10	10.0% [7.7-11.8]	2.3% [1.3-5.0]	3.3% [2.4-5.5]	11.4% [8.2-13.2]	4.3% [2.5-6.7]	6.0% [4.6-7.9]
	SB15	19.6% [17.1-20.9]	5.4% [4.3-8.7]	7.7% [5.9-12.2]	21.3% [17.8-24.0]	6.8% [5.3-8.5]	11.1% [9.3-13.6]
	SB20	32.2% [31.3-34.1]	9.6% [7.0-15.3]	12.6% [9.7-18.3]	34.0% [32.2-35.2]	12.0% [9.4-14.0]	17.7% [15.3-19.5]
	UB30	46.6% [45.6-47.3]	19.5% [14.4-26.9]	20.3% [15.6-27.3]	45.5% [43.2-46.6]	22.2% [13.6-27.1]	23.4% [15.3-27.4]
	UB40	48.9% [48.3-49.2]	21.6% [17.5-26.5]	21.8% [18.2-26.5]	48.5% [47.3-49.2]	25.0% [16.7-29.8]	26.9% [20.1-30.6]
	Biennial	49.1% [48.6-49.3]	22.4% [18.3-28.1]	22.7% [19.1-28.1]	49.1% [48.7-49.4]	25.1% [19.0-28.3]	27.6% [21.4-31.2]
20-30 PY	Annual	0.0% [0.0-0.0]	0.0% [0.0-0.0]	0.0% [0.0-0.0]	0.0% [0.0-0.0]	0.0% [0.0-0.0]	0.0% [0.0-0.0]
	EB10	8.2% [6.9-9.9]	9.8% [8.9-12.0]	6.3% [3.2-8.1]	6.2% [5.5-6.9]	9.8% [8.5-11.6]	5.3% [4.2-6.9]
	MA10	32.8% [31.6-33.8]	13.4% [11.7-17.5]	13.2% [12.0-17.6]	33.3% [31.3-34.8]	14.7% [12.2-17.9]	14.0% [10.4-18.8]
	MB10	15.4% [14.2-16.9]	7.9% [6.9-9.5]	8.7% [7.9-10.6]	14.9% [13.0-17.2]	8.6% [5.8-12.4]	8.8% [6.8-12.8]
	SB10	24.6% [21.7-26.9]	4.2% [3.4-5.5]	6.3% [5.2-9.5]	27.1% [24.4-29.3]	5.3% [3.9-7.4]	7.7% [5.6-10.2]
	SB15	33.0% [32.3-33.7]	7.8% [6.5-10.8]	11.3% [9.2-16.4]	35.9% [35.6-36.2]	9.3% [8.2-11.2]	12.4% [10.3-16.1]
	SB20	40.0% [38.6-41.1]	11.4% [9.4-15.3]	14.8% [12.4-20.6]	42.0% [41.6-42.3]	14.6% [12.2-18.9]	17.6% [13.6-23.3]
	UB30	30.5% [30.0-31.5]	7.3% [6.8-8.3]	9.4% [8.6-11.8]	32.9% [31.0-34.4]	9.2% [8.1-10.7]	10.8% [8.8-13.1]
	UB40	45.8% [45.2-46.4]	18.1% [16.1-22.3]	18.9% [16.8-25.2]	45.0% [44.5-45.3]	18.2% [15.4-22.6]	19.4% [15.2-25.3]
	Biennial	48.3% [48.0-48.5]	21.6% [18.2-27.8]	21.7% [18.7-29.0]	48.2% [47.8-48.5]	24.5% [19.4-30.5]	23.6% [18.3-30.2]
30+ PY	Annual	0.0% [0.0-0.0]	0.0% [0.0-0.0]	0.0% [0.0-0.0]	0.0% [0.0-0.0]	0.0% [0.0-0.0]	0.0% [0.0-0.0]
	EB10	9.3% [7.9-11.2]	9.7% [9.1-10.8]	6.7% [5.8-7.0]	8.4% [6.8-10.1]	9.1% [8.3-10.5]	6.3% [6.3-6.5]
	MA10	31.1% [31.1-31.2]	13.9% [12.5-16.1]	13.2% [11.7-15.9]	31.3% [31.0-31.5]	14.6% [10.5-18.5]	14.9% [10.4-20.0]
	MB10	17.3% [17.0-17.4]	7.9% [6.9-9.8]	8.7% [7.5-11.2]	17.1% [16.7-17.6]	8.9% [5.6-12.2]	10.1% [7.2-13.1]
	SB10	21.8% [20.0-23.2]	4.0% [3.0-6.2]	6.3% [4.7-9.3]	22.9% [20.9-24.7]	4.6% [1.8-7.1]	7.8% [4.1-11.1]
	SB15	30.8% [30.4-31.2]	7.2% [6.6-8.7]	10.4% [8.9-13.2]	32.2% [31.7-32.6]	8.1% [4.8-11.1]	12.6% [8.0-16.8]
	SB20	39.1% [37.4-40.3]	12.4% [9.9-16.2]	15.4% [12.3-20.0]	40.0% [38.5-41.3]	14.7% [8.4-20.7]	18.5% [11.7-24.3]

© 2025 de Nijs K et al. *JAMA Network Open*.

	UB30	0.2%<br>[0.0-0.4]	0.0%<br>[-0.2-0.0]	0.2%<br>[0.0-0.9]	0.2%<br>[0.0-0.4]	0.4%<br>[0.0-1.6]	0.4%<br>[0.0-1.1]
	UB40	18.4%<br>[17.8-18.9]	3.6%<br>[3.1-4.7]	4.7%<br>[4.1-6.3]	15.5%<br>[13.6-17.2]	2.8%<br>[2.0-3.9]	4.4%<br>[2.7-6.0]
	Biennial	48.4%<br>[48.2-48.6]	22.2%<br>[19.4-25.4]	22.2%<br>[19.4-25.7]	48.4%<br>[48.2-48.6]	23.6%<br>[17.0-30.3]	24.6%<br>[18.4-30.9]

Supplementary Table 5 reports the reductions in CT burden, deaths prevented and life years gained when moving from annual screening to various age-adaptive screening schedules (strategies are specified by their name per Table 1). Results are reported separately for those with 10-20, 20-30 and 30+ PY at age 50, and by biological sex. The numbers in brackets report the inter-model range of the estimates from each of three CISNET microsimulation models, specified in Supplementary Table 1.

© 2025 de Nijs K et al. *JAMA Network Open*.

eTable 6. Financial Cost, QALY, and LY Gained of All Strategies

	QALY Gained*	LY Gained*	Cost* ($)	ICER ($)	ICER($) excl. UB30/40	ACER ($)	CT Scans	CT Scan Reduction†	LC Deaths Prevented†	LY Gain†
No Screening	-	-	-	-	-		0k	100.0%	100.0%	100.0%
Biennial	8,702	13,622	312m	35,886	35,886	35,886	651k	48.5%	23.0%	23.4%
SB20	9,329	14,677	349m	-	58,946	37,436	778k	38.5%	12.9%	16.4%
MA10	9,651	15,339	381m	-	-	39,521	877k	30.7%	14.2%	13.8%
SB15	9,840	15,586	383m	-	65,713	38,904	888k	29.8%	7.5%	11.1%
UB40	10,138	16,081	397m	58,915	-	39,147	888k	29.8%	8.6%	9.5%
SB10	10,311	16,438	419m	-	76,600	40,623	1004k	20.6%	4.1%	6.6%
MB10	10,004	16,150	440m	-	-	43,962	1038k	17.9%	8.3%	9.5%
UB30	10,556	16,958	448m	123,379	-	42,481	1051k	16.9%	4.8%	4.9%
EB10	10,309	16,788	472m	-	-	45,803	1137k	10.1%	9.9%	6.9%
Annual	10,927	17,831	509m	162,857	146,050	46,576	1264k	-	-	-

Supplementary Table 6 shows the QALY gained, LY gained and cost of each strategy studied, scaled per 100,000 individuals alive at age 45 from the 1965 birth cohort. Estimates represent averages across the three microsimulation models. Strategies are shown sorted by increasing cost. From the QALY and cost values, the ICER is reported for the most cost-effective strategies. These represent the ICERS for the entire population, as reported in the final row of Table 3 in the main manuscript. For all strategies, the ACER (Average Cost-Effectiveness Ratio), that is the cost per QALY relative to no screening is reported. A discount rate of 3% is maintained. *Relative to no screening. †Reduction relative to annual screening.

© 2025 de Nijs K et al. *JAMA Network Open*.

eTable 7. Benefits and Harms of Age-Adaptive Screening Strategies

A. THE ERASMUS-MISCAN MODEL

Strategy Name*	CT Scans	CT Scans (%Red. From annual)	LC Screen-Detected Cases	LC Clinical Cases	LC Deaths	LC Deaths Prevented	LC Deaths Prevented (%Red. from annual)	LY Gain	LY Gain (%Red. from annual)	Excess Follow-up CT	Excess Biopsies	LC Cases	Over-diagnosed cases†	CT Screens/ Death Prevented
No Screening	0k	100.0%	-	11,303	8,786	-	100.0%	-	100.0%	-	-	11,303	0.0%	-
Biennial	605k	48.5%	3,327	8,161	7,373	1,413	27.8%	20,068	28.5%	74,448	2,555	11,488	5.6%	428
Annual	1174k	0.0%	4,247	7,309	6,827	1,959	0.0%	28,050	0.0%	136,683	4,800	11,556	6.0%	599
SB10	929k	20.9%	4,105	7,446	6,925	1,861	5.0%	25,740	8.2%	109,525	3,822	11,552	6.1%	499
SB15	835k	28.9%	3,999	7,550	6,994	1,792	8.5%	24,450	12.8%	99,224	3,452	11,549	6.2%	466
SB20	719k	38.8%	3,771	7,767	7,137	1,649	15.8%	22,286	20.5%	86,735	3,000	11,538	6.2%	436
EB10	1060k	9.7%	3,823	7,684	7,055	1,731	11.6%	25,933	7.5%	124,399	4,355	11,506	5.3%	612
MA10	814k	30.6%	3,681	7,822	7,152	1,634	16.6%	23,623	15.8%	97,240	3,377	11,502	5.4%	498
MB10	964k	17.9%	3,923	7,619	7,035	1,751	10.6%	24,678	12.0%	113,895	3,977	11,542	6.1%	551
UB30	978k	16.7%	4,062	7,482	6,933	1,853	5.4%	26,496	5.5%	114,462	4,010	11,544	5.9%	528
UB40	820k	30.2%	3,919	7,614	7,019	1,766	9.8%	25,018	10.8%	97,505	3,392	11,533	5.9%	464

*Supplementary Table 7A reports summary outcomes of age-adaptive screening strategies, as generated by the Erasmus MISCAN model. For model summaries, see Supplementary Table 1. The outcomes are scaled per 100,000 individuals alive at age 45, with a minimum smoking exposure of 10PY at age 50 (some of whom may become eligible at a later age as they accumulate PY). *Strategy name per Table 1 † Overdiagnosed lung cancer cases, given by the share of screen-detected lung-cancers that would not have presented clinically in a scenario without screening.*

© 2025 de Nijs K et al. *JAMA Network Open*.

B. THE BCCRI-LUNGCAN MODEL

Strategy Name*	CT Scans	CT Scans (%Red. From annual)	LC Screen-Detected Cases	LC Clinical Cases	LC Deaths	LC Deaths Prevented	LC Deaths Prevented (%Red. from annual)	LY Gain	LY Gain (%Red. from annual)	Excess Follow-up CT	Excess Biopsies	LC Cases	Over-diagnosed cases†	CT Screens/ Death Prevented
No Screening	0k	100.0%	-	15,754	13,125	-	100.0%	-	100.0%	-	-	15,754	0.0%	-
Biennial	600k	48.4%	4,566	11,434	11,875	1,251	27.0%	22,780	27.7%	26,350	3,487	16,000	5.4%	480
Annual	1163k	0.0%	5,941	10,107	11,413	1,712	0.0%	31,513	0.0%	46,097	5,165	16,048	4.9%	679
SB10	909k	21.9%	5,707	10,352	11,524	1,601	6.5%	28,539	9,4%	37,495	4,645	16,059	5.4%	567
SB15	816k	29.9%	5,513	10,532	11,578	1,548	9.6%	26,861	14.8%	34,277	4,350	16,046	5.3%	527
SB20	704k	39.5%	5,196	10,848	11,711	1,415	17.4%	24,787	21.3%	30,217	3,978	16,044	5.6%	498
EB10	1060k	8.9%	5,353	10,671	11,584	1,542	10.0%	29,388	6.7%	42,370	4,685	16,024	5.0%	687
MA10	805k	30.9%	5,084	10,923	11,700	1,425	16.8%	26,142	17.0%	33,592	4,108	16,006	5.0%	564
MB10	959k	17.6%	5,464	10,579	11,573	1,552	9.4%	28,161	10.6%	39,006	4,549	16,043	5.3%	618
UB30	961k	17.4%	5,654	10,394	11,532	1,594	6.9%	29,357	6.8%	39,180	4,669	16,048	5.2%	603
UB40	812k	30.2%	5,429	10,602	11,593	1,533	10.5%	27,766	11.9%	34,102	4,271	16,030	5.1%	530

*Supplementary Table 7B reports summary outcomes of age-adaptive screening strategies, as generated by the University of Michigan Model. For model summaries, see Supplementary Table 1. The outcomes are scaled per 100,000 individuals alive at age 45, with a minimum smoking exposure of 10PY at age 50 (some of whom may become eligible at a later age as they accumulate PY). *Strategy name per Table 1 † Overdiagnosed lung cancer cases, given by the share of screen-detected lung-cancers that would not have presented clinically in a scenario without screening.*

© 2025 de Nijs K et al. *JAMA Network Open*.

C. THE ONCOSIM MODEL

Strategy Name*	CT Scans	CT Scans (%Red. From annual)	LC Screen-Detected Cases	LC Clinical Cases	LC Deaths	LC Deaths Prevented	LC Deaths Prevented (%Red. from annual)	LY Gain	LY Gain (%Red. from annual)	Excess Follow-up CT	Excess Biopsies	LC Cases	Over-diagnosed cases†	CT Screens/ Death Prevented
No Screening	0k	100.0%	-	27,694	12,923	-	100.0%	-	100.0%	-	-	27,694	0.0%	-
Biennial	749k	48.6%	7,139	20,920	10,073	2,850	18.4%	50,180	19.0%	10,381	1,974	28,060	5.1%	263
Annual	1456k	0.0%	8,455	19,800	9,432	3,491	0.0%	61,925	0.0%	16,252	3,285	28,255	6.6%	417
SB10	1174k	19.3%	8,232	19,981	9,521	3,402	2.5%	59,202	4.4%	14,217	2,798	28,213	6.3%	345
SB15	1014k	30.3%	7,993	20,183	9,635	3,288	5.8%	56,690	8.5%	12,963	2,507	28,176	6.0%	308
SB20	911k	37.4%	7,764	20,368	9,751	3,172	9.1%	54,492	12.0%	12,046	2,310	28,132	5.6%	287
EB10	1292k	11.3%	7,841	20,348	9,747	3,176	9.0%	57,740	6.8%	14,568	2,938	28,189	6.3%	407
MA10	1011k	30.6%	7,626	20,519	9,839	3,084	11.6%	55,001	11.2%	12,510	2,450	28,145	5.9%	328
MB10	1191k	18.2%	7,977	20,181	9,658	3,265	6.5%	57,140	7.7%	14,113	2,790	28,158	5.8%	365
UB30	1214k	16.6%	8,216	20,004	9,552	3,371	3.4%	59,629	3.7%	14,469	2,867	28,220	6.4%	360
UB40	1032k	29.1%	7,951	20,233	9,679	3,244	7.1%	57,122	7.8%	13,022	2,526	28,184	6.2%	318

*Supplementary Table 7C reports summary outcomes of age-adaptive screening strategies, as generated by the OncoSim Model. For model summaries, see Supplementary Table 1. The outcomes are scaled per 100,000 individuals alive at age 45, with a minimum smoking exposure of 10PY at age 50 (some of whom may become eligible at a later age as they accumulate PY). *Strategy name per Table 1 † Overdiagnosed lung cancer cases, given by the share of screen-detected lung-cancers that would not have presented clinically in a scenario without screening.*

© 2025 de Nijs K et al. *JAMA Network Open*.

eTable 8. Cost-Effective Age-Adaptive Screening Strategies at Increasing WTP Thresholds by Risk Stratum (in Pack-Years at 50 Years of Age) and Biological Sex, Excluding Strategies That Require Updated PY Information

		WTP Threshold				
Sex	Risk Stratum (PY at age 50)	$50,000	$75,000	$100,000	$150,000	$200,000
Male	10-20 PY	No Screening*	Biennial ($55,527)	Biennial ($55,527)	Annual ($146,267)	Annual ($146,267)
Male	20-30 PY	No Screening*	Biennial ($56,511)	SB15 ($85,231)	SB10 ($140,505)	SB10 ($140,505)
Male	30+ PY	Biennial ($35,826)	SB10 ($70,640)	SB10 ($70,640)	Annual ($125,573)	Annual ($125,573)
Male	All	Biennial ($40,809)	SB15 ($68,436)	SB10 ($87,072)	SB10 ($87,072)	Annual ($157,027)
Female	10-20 PY	Biennial ($40,105)	Biennial ($40,105)	SB20 ($83,184)	SB10 ($122,392)	SB10 ($122,392)
Female	20-30 PY	Biennial ($38,599)	SB20 ($55,160)	SB10 ($80,329)	SB10 ($80,329)	SB10 ($80,329)
Female	30+ PY	SB15 ($49,994)	SB10 ($57,023)	SB10 ($57,023)	Annual ($103,875)	Annual ($103,875)
Female	All	Biennial ($31,934)	SB10 ($67,306)	SB10 ($67,306)	Annual ($133,709)	Annual ($133,709)
All	10-20 PY	Biennial ($45,692)	Biennial ($45,692)	SB20 ($89,885)	SB10 ($131,668)	Annual ($170,744)
All	20-30 PY	Biennial ($45,478)	SB20 ($62,015)	SB10 ($95,173)	SB10 ($95,173)	SB10 ($95,173)
All	30+ PY	SB20 ($48,568)	SB10 ($63,257)	SB10 ($63,257)	Annual ($114,016)	Annual ($114,016)
All	All	Biennial ($35,886)	SB15 ($65,713)	SB10 ($76,600)	Annual ($146,050)	Annual ($146,050)

Supplementary Table 8 reports for different risk strata the cost-efficient age-adaptive lung cancer screening strategy at increasing willingness-to-pay thresholds, as well as the Incremental Cost-Effectiveness Ratio (the incremental cost per Quality-Adjusted Life Year (QALY) gained) of that strategy relative to the strategy preceding it on the efficient frontier (the most efficient strategy yielding fewer total QALYs). Strategy characteristics by name are given in Table 1. The risk stratum based on smoking exposure is determined by the number of PY at age 50. For the lowest risk stratum, some may start screening as they cross the 20 PY minimum set by the 2021 USPSTF guidelines, while some may never be screened. For this set of efficient frontiers, we only consider age-adaptive screening strategies, as opposed to Table 3 in the main text, where strategies UB30 and UB40 are included, which update the interval based on the cumulative smoking exposure in PY. *"No screening" indicates that no strategy studied had an ICER below the WTP threshold.

eTable 9. Benefits and Harms of Age-Adaptive Screening Strategies, Assuming American Cancer Society Eligibility

Strategy Name*	CT Scans	CT Scans (%Red. From annual)	LC Screen-Detected Cases	LC Clinical Cases	LC Deaths	LC Deaths Prevented	LC Deaths Prevented (%Red. from annual)	LY Gain	LY Gain (%Red. from annual)	Excess Follow-up CT	Excess Biopsies	LC Cases	Over-diagnosed cases†	CT Screens/ Death Prevented
No Screening	0k	100.0%	-	18,258	11,617	-	100.0%	-	100.0%	-	-	18,258	0.0%	-
Biennial	856k	48.5%	6,032	12,538	9,433	2,184	22.9%	35,534	23.3%	48,966	3,454	18,570	5.2%	392
Annual	1662k	0.0%	7,453	11,241	8,783	2,834	0.0%	46,324	0.0%	88,995	5,754	18,694	5.8%	586
SB10	1380k	17.0%	7,250	11,431	8,879	2,738	3.4%	43,646	5.8%	75,082	5,025	18,681	5.8%	504
SB15	1243k	25.2%	7,070	11,590	8,971	2,647	6.6%	41,744	9.9%	69,007	4,667	18,661	5.7%	470
SB20	1091k	34.3%	6,793	11,845	9,103	2,515	11.3%	39,491	14.8%	60,766	4,199	18,638	5.6%	434
EB10	1426k	14.2%	6,740	11,890	9,098	2,520	11.1%	42,690	7.8%	77,272	5,005	18,630	5.5%	566
MA10	1144k	31.1%	6,526	12,085	9,200	2,417	14.7%	39,896	13.9%	63,329	4,277	18,612	5.4%	473
MB10	1373k	17.4%	7,011	11,639	8,999	2,618	7.6%	42,239	8.8%	74,617	4,952	18,650	5.6%	524
UB30	1340k	19.4%	7,140	11,535	8,936	2,681	5.4%	43,812	5.4%	72,848	4,892	18,674	5.8%	500
UB40	1126k	32.2%	6,867	11,786	9,062	2,555	9.8%	41,520	10.4%	62,129	4,308	18,653	5.7%	441
UB15cess	1550k	6.7%	7,320	11,364	8,855	2,762	2.6%	45,382	2.0%	80,999	5,380	18,683	5.8%	561

*Supplementary Table 9 reports summary outcomes of age-adaptive screening strategies, if applied to all those with 20 PY, but no limit on the years since smoking cessation, as per the American Cancer Society Guidelines.[9] Outcomes are scaled per 100,000 individuals alive at age 45, with a minimum smoking exposure of 10PY at age 50 (some of whom may become eligible from a later age as they accumulate PY). *Strategy name per Table 1 † Overdiagnosed lung cancer cases, given by the share of screen-detected lung-cancers that would not have presented clinically in a scenario without screening. ‡ Number Needed to Screen (NNS) per death prevented, reports the number of CT scans required to yield one death prevented.*

© 2025 de Nijs K et al. *JAMA Network Open*.

eTable 10. Cost-Effective Age-Adaptive Screening Strategies at Increasing WTP Thresholds by Risk Stratum and Biological Sex, per American Cancer Society Guidelines

		WTP Threshold				
Sex	Risk Stratum (PY at age 50)	$50,000	$75,000	$100,000	$150,000	$200,000
Male	10-20 PY	No Screening*	Biennial ($58,540)	Biennial ($58,540)	Biennial ($58,540)	UB15cess ($156,191)
Male	20-30 PY	No Screening*	Biennial ($68,090)	UB40 ($88,851)	UB30 ($103,957)	UB30 ($103,957)
Male	30+ PY	Biennial ($38,495)	SB20 ($62,165)	UB40 ($75,823)	UB40 ($75,823)	UB30 ($174,834)
Male	All	Biennial ($45,212)	UB40 ($70,525)	UB40 ($70,525)	UB30 ($144,869)	UB30 ($144,869)
Female	10-20 PY	UB40 ($40,801)	UB40 ($40,801)	UB40 ($40,801)	SB10 ($145,410)	UB15cess ($169,575)
Female	20-30 PY	UB40 ($45,655)	UB40 ($45,655)	UB30 ($78,884)	UB30 ($78,884)	UB30 ($78,884)
Female	30+ PY	Biennial ($30,783)	UB40 ($53,962)	UB40 ($53,962)	Annual ($131,460)	Annual ($131,460)
Female	All	Biennial ($34,605)	UB40 ($54,080)	UB40 ($54,080)	UB30 ($122,046)	Annual ($180,952)
All	10-20 PY	Biennial ($46,948)	UB30 ($65,167)	UB30 ($65,167)	SB15 ($127,441)	UB15cess ($153,362)
All	20-30 PY	No Screening*	UB40 ($59,669)	UB30 ($87,819)	UB30 ($87,819)	UB30 ($87,819)
All	30+ PY	Biennial ($34,063)	UB40 ($63,978)	UB40 ($63,978)	UB40 ($63,978)	Annual ($152,231)
All	All	Biennial ($39,305)	UB40 ($62,277)	UB40 ($62,277)	UB30 ($131,929)	UB30 ($131,929)

Supplementary Table 10 reports for different risk strata the cost-efficient age-adaptive lung cancer screening strategy at increasing willingness-to-pay thresholds, as well as the Incremental Cost-Effectiveness Ratio (the incremental cost per Quality-Adjusted Life Year (QALY) gained) of that strategy relative to the strategy preceding it on the efficient frontier (the most efficient strategy yielding fewer total QALYs). Strategy characteristics by name are given in Table 1. The risk stratum based on smoking exposure is determined by the number of PY at age 50. For the lowest risk stratum, some may start screening as they cross the 20 PY minimum set by the USPSTF2021 guidelines, while some may never be screened. For this set of efficient frontiers, it is assumed no limit is set to the maximal years since smoking cessation, as opposed to table 3, where screening is offered only to those with less than 15 years since cessation. *"No screening" indicates that no strategy studied had an ICER below the WTP threshold. Cells are color-coded to biennial screening (orange), adaptive screening (purple), annual screening (blue).

© 2025 de Nijs K et al. *JAMA Network Open*.

eFigure 1. Model Structure Diagram

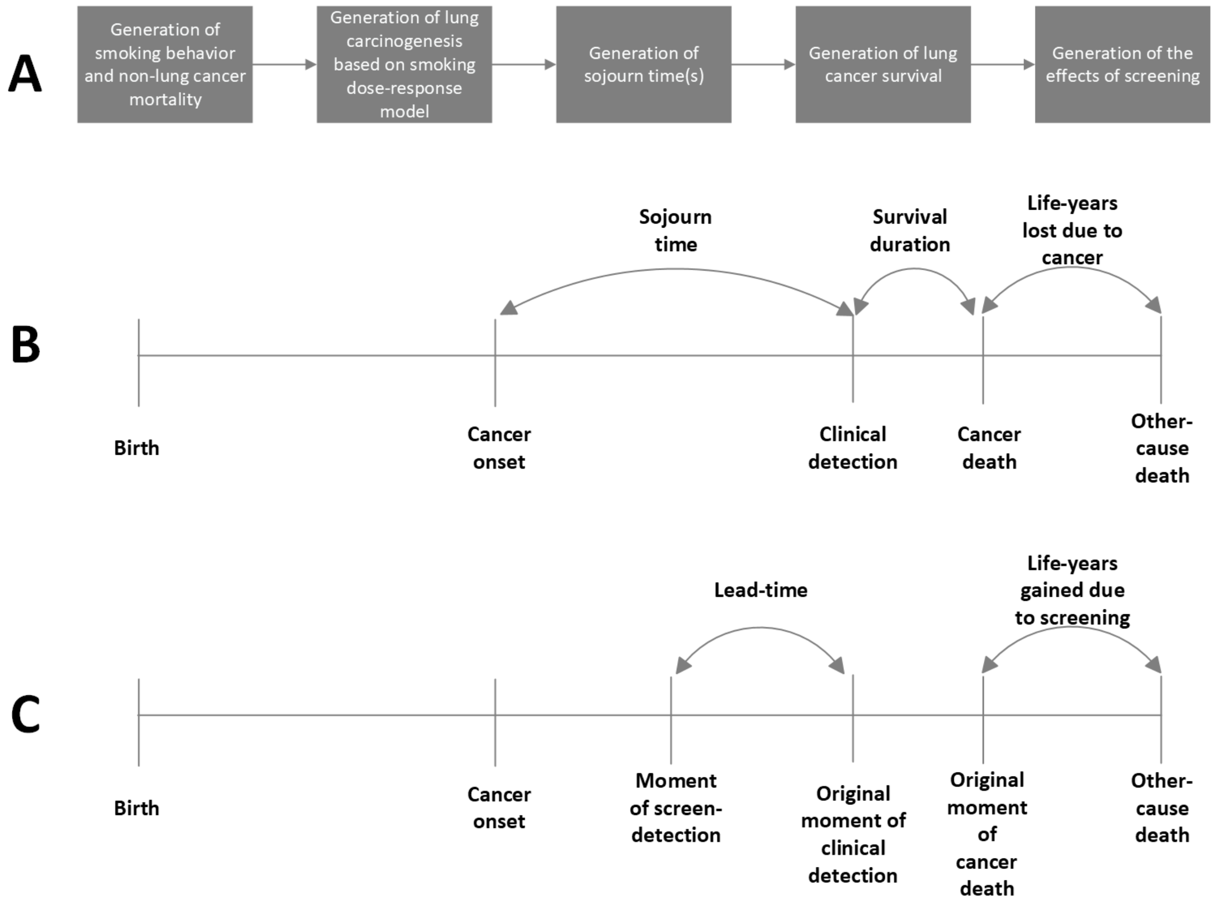

Supplementary Figure 1 (reproduced from ten Haaf et al, MDM, 2024) demonstrates the general mechanics of the microsimulation models employed in this study. **Part A** shows the steps in which a lung cancer is generated from simulated smoking behaviours. The age of cancer onset, as well as the age of death from non-lung cancer causes are drawn randomly, but depend on the history of smoking of the individual. **Part B** shows a non-screening scenario; the cancer spends a period undetected (the sojourn time) before being detected symptomatically. After detection, the length of survival is drawn from model-specific survival curves. **Part C** shows a scenario with screening; a screening event may lead to early detection. The early detection may result in a cure or a prolonging on survival, depending on the mechanics of the model. This may result in life years gained from screening if the individual would have otherwise died from lung cancer.

© 2025 de Nijs K et al. *JAMA Network Open*.

eFigure 2. Life-Years Gained and Reduction in CT Scans Required for Annual, Biennial, and Age-Adaptive (Starting With Biennial) Screening

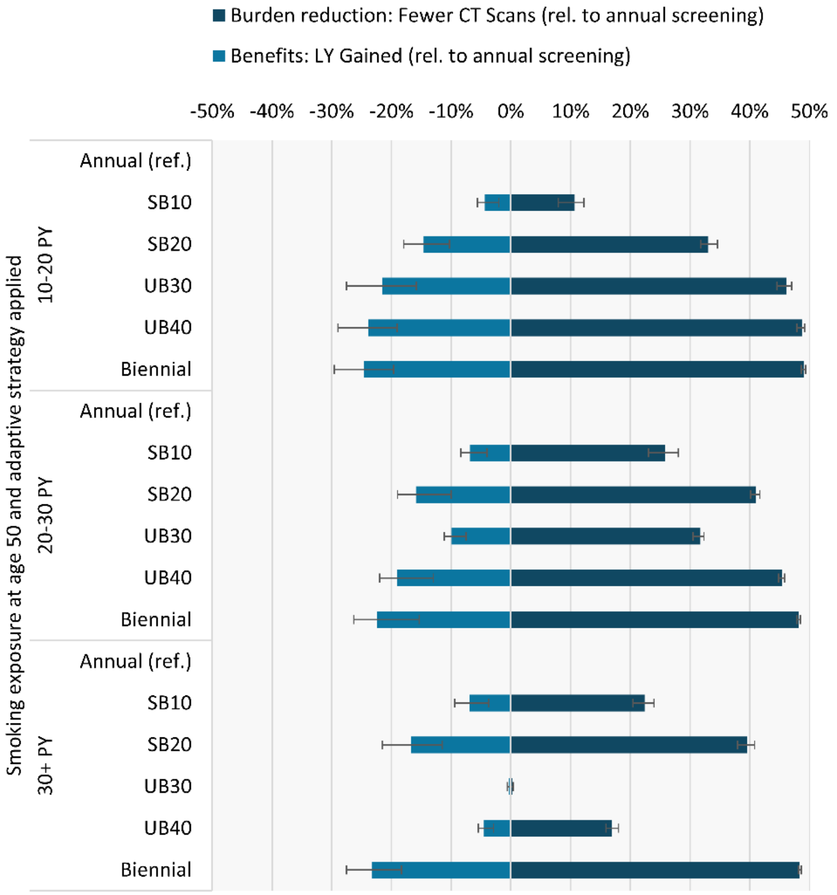

**SB10: Biennial screening ages 50-59 followed by annual, SB20: Biennial screening ages 50-69 followed by annual, UB30: Biennial screening, annual from 30 pack-years, UB40: Biennial screening, annual from 40 pack-years*

Supplementary Figure 2 shows reductions in life years gained and required CT volume, relative to annual screening, for biennial screening and screening that starts with10- or 20 years of biennial screening before annual screening. Error bars represent the inter-model range of the results across the 3 microsimulation models included in our study. This figure pertains to males and females of increasing lung cancer risk categories: 10-20PY, 20-30PY and 30+ PY, at age 50. Those with 10-20PY start screening if they cross the USPSTF2021 threshold of 20PY. The full input data to this figure, as well as the data for strategies not shown here, are presented in Supplementary Table 5.

© 2025 de Nijs K et al. *JAMA Network Open*.

eFigure 3. Cost-Effective Frontier of Age-Adaptive Screening Strategies by Sex and Risk Stratum

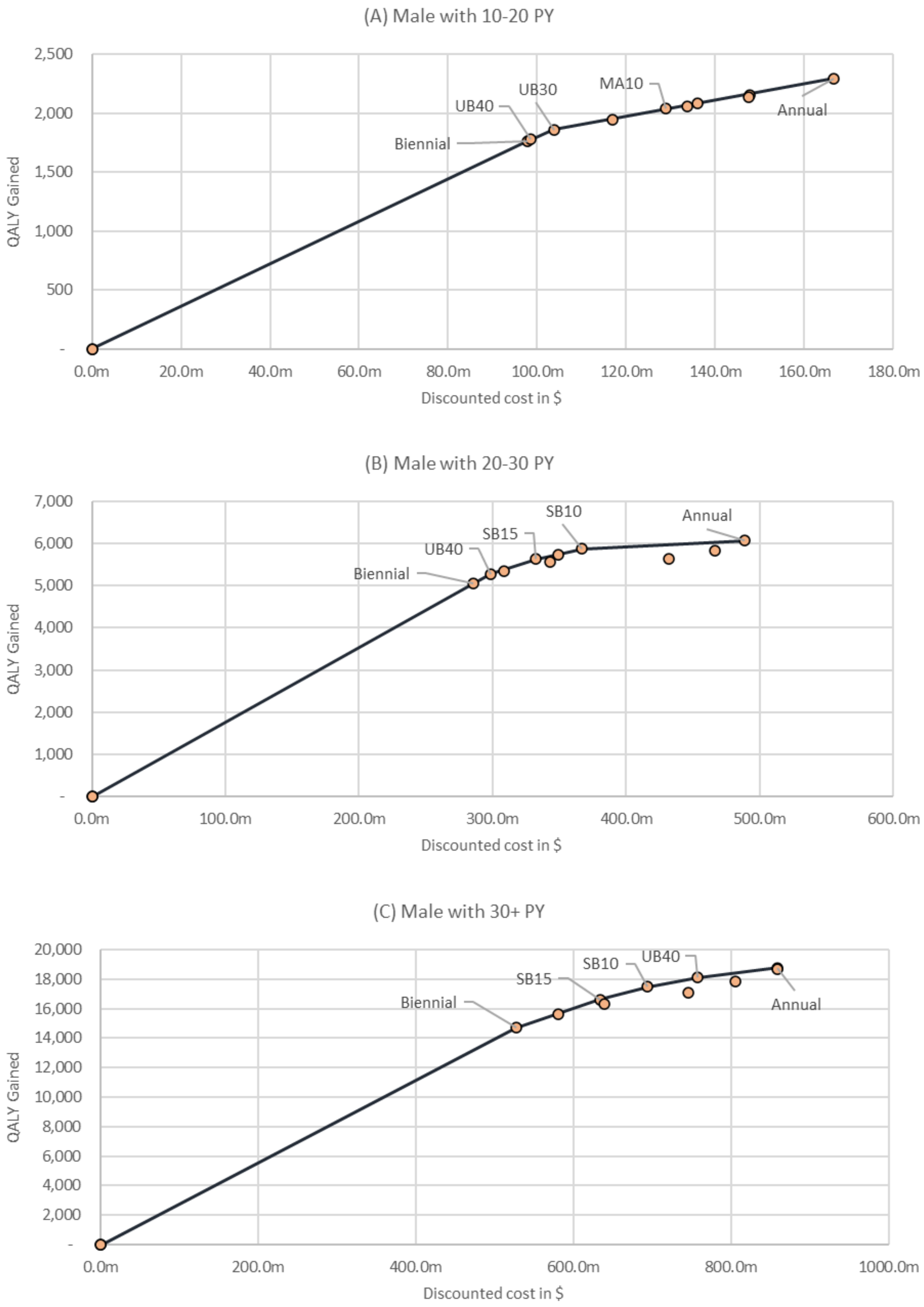

© 2025 de Nijs K et al. *JAMA Network Open*.

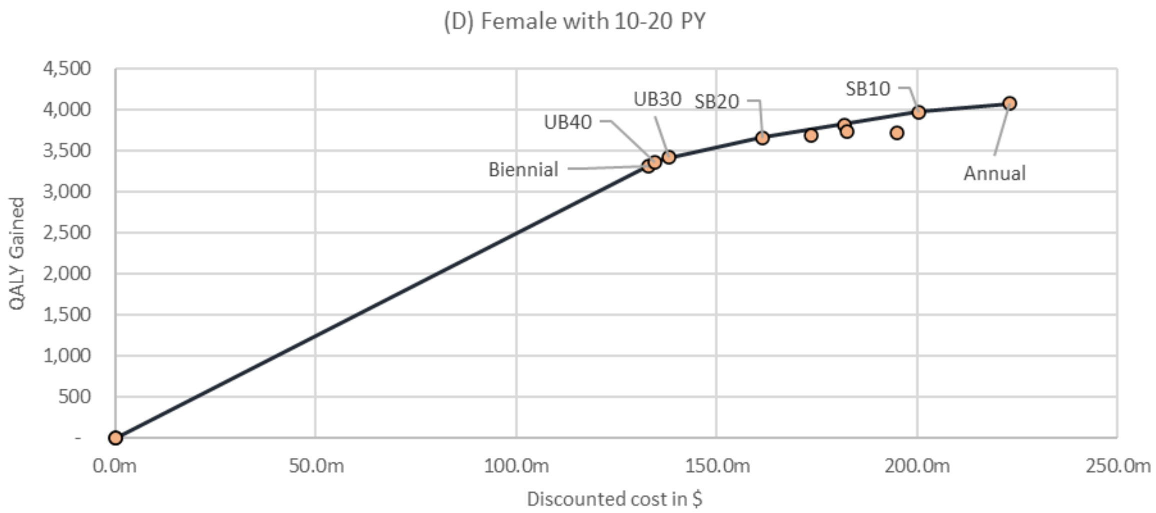

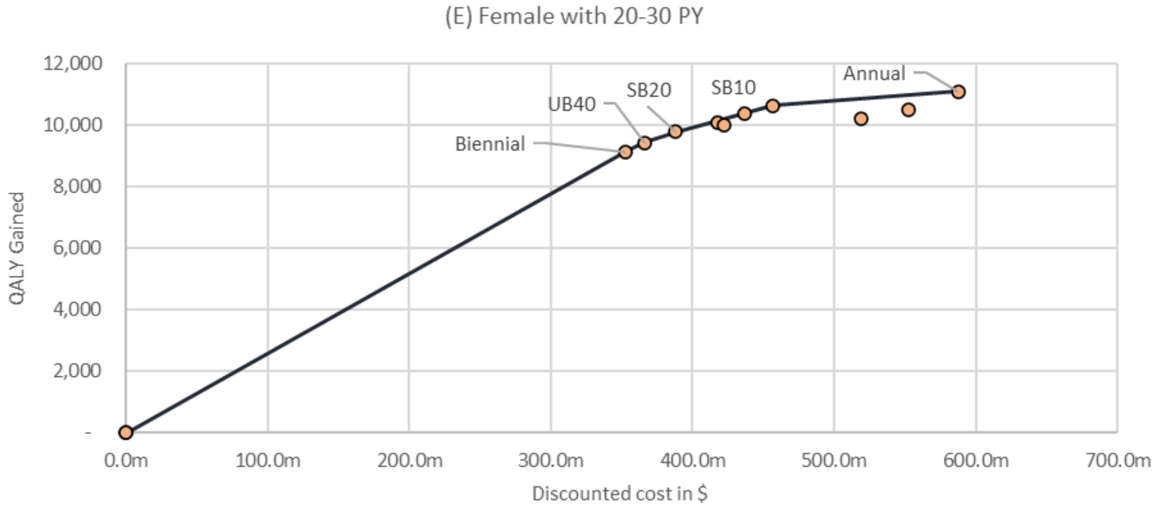

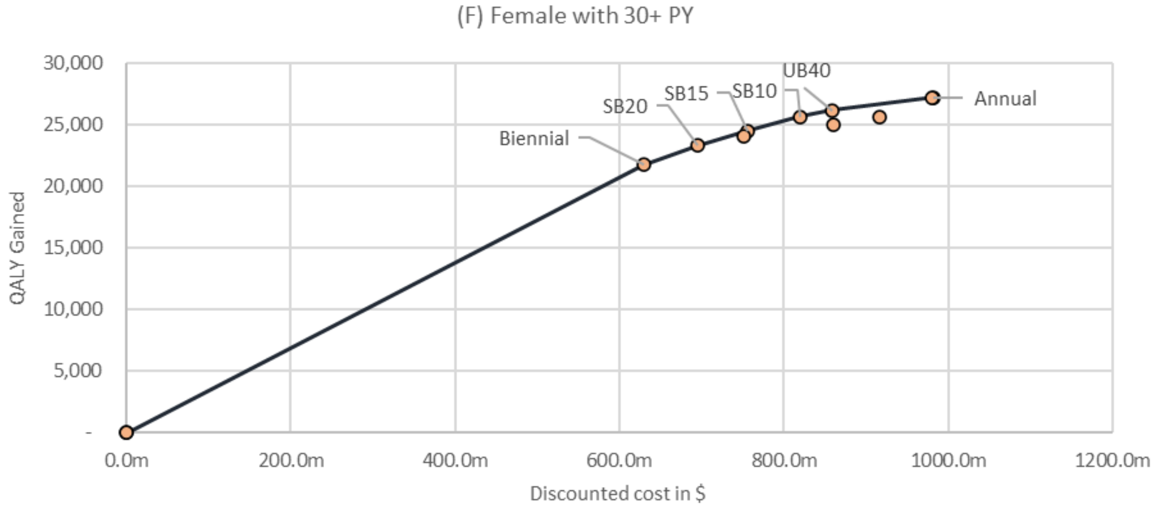

© 2025 de Nijs K et al. *JAMA Network Open*.

Supplementary Figure 3 (A) through (F) shows the effective frontier of age-adaptive strategies for each risk group (10-20 PY, 20-30 PY, 30+ PY at age 50) and sex. The costs and quality adjusted life years (QALYs) for each screening strategy is reported relative to a no-screening scenario, scaled to 100,000 individuals alive at age 45 (of the specific risk group). Cost and QALYs are discounted by 3% from age 50. Strategy names are reported for those strategies on the efficient frontier, per the strategy names reported in Table 1 of the main text.

© 2025 de Nijs K et al. *JAMA Network Open*.

eReferences

1. Hanmer J, Lawrence WF, Anderson JP, et al. Report of nationally representative values for the noninstitutionalized US adult population for 7 health-related quality-of-life scores. *Med Decis Making* 2006;26:391-400.
2. Tramontano AC, Schrag DL, Malin JK, et al. Catalog and comparison of societal preferences (utilities) for lung cancer health states: results from the Cancer Care Outcomes Research and Surveillance (CanCORS) study. *Med Decis Making* 2015;35:371-387.
3. McMahon PM, Kong CY, Bouzan C, et al. Cost-effectiveness of computed tomography screening for lung cancer in the United States. *J Thorac Oncol* 2011;6:1841-1848.
4. Caverly TJ, Hayward RA, Meza R. Identifying Patients for Whom Lung Cancer Screening Is Preference-Sensitive. *Ann Intern Med* 2018;169:823.
5. Toumazis I, Tsai EB, Erdogan SA, et al. Cost-Effectiveness Analysis of Lung Cancer Screening Accounting for the Effect of Indeterminate Findings. *JNCI Cancer Spectr* 2019;3:pkz035.
6. Sheehan DF, Criss SD, Chen Y, et al. Lung cancer costs by treatment strategy and phase of care among patients enrolled in Medicare. *Cancer Med* 2019;8:94-103.
7. Toumazis I, Cao P, de Nijs K, et al. Risk Model-Based Lung Cancer Screening : A Cost-Effectiveness Analysis. *Ann Intern Med* 2023;176:320-332.
8. Consumer price index (2010=100 - United States. In: International Monetary Fund, ed. *International Financial Statistics and data files,*. World Bank; 2023 Available at https://data.worldbank.org/indicator/FP.CPI.TOTL. Accessed 01-10-2024.
9. Wolf AMD, Oeffinger KC, Shih TY, et al. Screening for lung cancer: 2023 guideline update from the American Cancer Society. *CA Cancer J Clin* 2024;74:50-81.

© 2025 de Nijs K et al. *JAMA Network Open*.
