# Supplementary material for: Computed Tomographic Screening Intervals for Patients at Moderate Risk of Lung Cancer
Source: JAMA Netw Open. 2025 Jul 24;8(7):e2523044. doi: 10.1001/jamanetworkopen.2025.23044 (PMC12290729; doi:10.1001/jamanetworkopen.2025.23044)
Supplement: Supplement 2. — Data Sharing Statement [file jamanetwopen-e2523044-s002.pdf]

Data Sharing Statement

de Nijs. Computed Tomographic Screening Intervals for Patients at Moderate Risk of Lung Cancer. *JAMA Netw Open*. Published July 24, 2025.
doi:10.1001/jamanetworkopen.2025.23044

Data
Data available: No
